# The Roles of Serum Selenium and Selenoproteins on Mercury Toxicity in Environmental and Occupational Exposure

**DOI:** 10.1289/ehp.7861

**Published:** 2006-01-18

**Authors:** Chunying Chen, Hongwei Yu, Jiujiang Zhao, Bai Li, Liya Qu, Shuiping Liu, Peiqun Zhang, Zhifang Chai

**Affiliations:** 1 Laboratory of Nuclear Analytical Techniques and Laboratory for Nanoscale Materials and Related Bio-Environmental Sciences, Institute of High Energy Physics, Chinese Academy of Sciences, Beijing, People’s Republic of China; 2 Guizhou Research and Designing Institute of Environmental Sciences, Guiyang, People’s Republic of China; 3 Wanshan Office of Environmental Protection, Guizhou, People’s Republic of China

**Keywords:** antagonism, Hg-exposed subjects, mercury, selenium, selenoproteins, serum

## Abstract

Many studies have found that mercury (Hg) exposure is associated with selenium (Se) accumulation *in vivo*. However, human studies are limited. To study the interaction between Se and Hg, we investigated the total Se and Hg concentrations in body fluids and serum Se-containing proteins in individuals exposed to high concentrations of Hg. Our objective was to elucidate the possible roles of serum Se and selenoproteins in transporting and binding Hg in human populations. We collected data from 72 subjects: 35 had very low Hg exposure as evidenced by mean Hg concentrations of 0.91 and 1.25 ng/mL measured in serum and urine, respectively; 37 had high exposure (mean Hg concentrations of 38.5 and 86.8 ng/mL measured in serum and urine, respectively). An association between Se and Hg was found in urine (*r* = 0.625; *p* < 0.001) but not in serum. Hg exposure may affect Se concentrations and selenoprotein distribution in human serum. Expression of both selenoprotein P and glutathione peroxidase (GSH-Px) was greatly increased in Hg miners. These increases were accompanied by elevated Se concentrations in serum. In addition, selenoprotein P bound more Hg at higher Hg exposure concentrations. Biochemical observations revealed that both GSH-Px activity and malondialdehyde concentrations increased in serum of the Hg-exposed group. This study aids in the understanding of the interaction between Se and Hg. Selenoproteins play two important roles in protecting against Hg toxicity. First, they may bind more Hg through their highly reactive selenol group, and second, their antioxidative properties help eliminate the reactive oxygen species induced by Hg *in vivo*.

Mercury (Hg) is currently one of the most prevalent pollutants in the environment. It is highly bioconcentrated through the food chain and damages mainly nerves and immune systems. It is harmful both to humans and animals (Clarkson 1997).

Selenium (Se) is an essential micronutrient with important biological and biochemical functions in organisms because of its unique antioxidant properties and its ability to regulate thyroid gland metabolism. It is well known that Se is an antagonist that moderates the toxic effects of many heavy metals such as arsenic, cadmium, Hg, and lead in organisms. Although Se and Hg co-accumulation in humans and other mammals is well known (Falnoga et al. 2000; Kosta et al. 1975), the mechanism of interaction between Se and Hg is still not understood. It is thought to be attributed to the formation of biologically inert Hg–Se compounds. Burk et al. (1974) suggested that the Hg–Se–protein complex plays a role in restraining the acute toxicity of inorganic Hg by binding Hg to prevent it from reaching the target tissues. Recent *in vitro* studies suggest that Se and Hg could form Hg–Se complexes in a reducing environment and that this 1:1 complex is then bound with plasma selenoprotein P (SeP) (Suzuki et al. 1998; Yoneda and Suzuki 1997).

In mammalian serum, Se is incorporated mainly into three proteins—SeP, extracellular glutathione peroxidase (GSH-Px), and albumin. The first two are well-known selenoproteins. Naturally occurring selenoproteins such as thioredoxin reductases, GSH-Px, SeP, iodothyronine deiodinase types I, II, and III, and others with specific functions have also been identified (Kryukov et al. 2003). Among them, SeP is a unique selenoprotein and contains several selenocysteine (Sec) and cysteine (Cys) residues, indicating that it is capable of transporting Se and binding heavy metals. The sequence of the cloned DNA shows that SeP contains 10 Sec groups encoded by UGA stop codons in the open reading frame of its mRNA (Burk et al. 2001; Ma et al. 2002). But purified SeP from humans and rats was recently characterized by immunoaffinity chromatography and was found to contain 7–8 Se atoms per Sec molecule as Se attached to a Cys base. On the basis of *in vitro* studies (Suzuki et al. 1998), it has been suggested that Se exhibits protective effects against Hg toxicity in humans because of formation of an Hg–Se complex bound to SeP in blood; however, this has not been demonstrated *in vivo* in human populations.

Thus, our aim in this study was to evaluate the relationship between Se or selenoproteins and Hg exposure in humans. First, we compared Se and Hg concentrations in serum and urine from highly Hg-exposed and control subjects to determine whether Hg exposure affects the distribution and absorption of Se. Second, we investigated the role of plasma selenoproteins in transporting or accumulating Hg, and finally, oxidative stress as evidenced by malondialdehyde (MDA) concentration and GSH-Px activity in the Hg-exposed population. We also attempted to elucidate the antagonism between Hg and Se.

## Materials and Methods

### Collection of samples.

We selected subjects from the town of Wanshan in the Guizhou Province in southwest China, which is representative of an Hg-contaminated region. Present global Hg emission into the atmosphere is estimated to be 5,000 tonnes per year (Chen et al. 2005). The environment polluted by Hg in Guizhou Province is typical of other areas in China, where Hg comes from several sources: Hg mining and ore processing, coal combustion for power production, and chlor-alkali industries. The main source of environmental Hg pollution in Wanshan is the emission of elemental Hg vapor from an Hg-mining plant that had produced large quantities of Hg for more than 50 years. The plant was closed in 2001.

In 2000 and 2003, serum and urine samples were collected from 37 individuals (including 25 miners and 12 local residents) from a heavily Hg-contaminated area. These individuals are the exposed group. Samples were also collected from 35 residents from a noncontaminated area. These individuals form the control group. Between the groups there were no differences in sex, age (28–66 years of age), body mass index, and physical activity. All participants volunteered and agreed to the test. This study was approved and supported by the local Committee of Human Subjects and the local hospital. Detailed handling of samples was performed as described elsewhere (Chen et al. 2005).

### Chemicals and equipment.

Reagents and solvents were at least of analytical grade. The Milli-Q system (Millipore, Bedford, MA, USA) was used to prepare ultrapure water. We used a dual-channel hydride generation-atomic fluorescence spectrometer (HG-AFS) with a quartz furnace atomizer (model AFS-820; Beijing Little Swan Co., Beijing, China) to determine Hg and Se concentrations.

### Measurement of Hg exposure.

Total Hg concentrations in urine and serum samples were determined by HG-AFS. Detailed procedures have been described elsewhere using a sodium tetrahydridoborate (NaBH_4_)-acid system to generate Hg vapor for AFS (Zhao et al. 2004). The detection limit was 0.05 μg/L Hg. Hg-certified NIST (National Institute of Standards and Technology, Gaithersburg, MD, USA) bovine liver 1577a, IAEA horse kidney H-8 (International Agency Atomic Energy Agency, Vienna, Austria), and Chinese bovine liver GBW 080193 (National Research Center for Certified Reference Materials, Beijing, China) were analyzed for Hg as quality control standards, with analytical errors less than ± 10%.

### Determination of Se concentration.

We measured Se using HG-AFS according to detailed procedures described elsewhere (Chen et al. 1999). Briefly, samples were digested with a mixture of ultrapure nitric acid and perchloric acid (3:1) at 100°C for 2–3 hr. One milliliter of 5 mol/L HCl was added to the clear digested sample solution for an additional 10 min to reduce the existing selenate(VI) to selenite(IV). The solutions were then diluted to 15 mL, introduced into an NaBH_4_-acid system to generate hydrogen selenide, and subjected to HG-AFS. Quality control standards were analyzed as described in “Measurement of Hg exposure.”

### Assay of GSH-Px activity and MDA.

GSH-Px activity was measured according to the method of Hafeman et al. (1974). One unit was defined as a decrease of the reduced log (GSH) of 1 μmol/min at 37°C, whereas heated samples with inactivated enzymes were used as a nonenzymatic control to eliminate interference from endogenous reduced GSH. Total protein concentration in human serum was quantitatively determined by the Bradford method using bovine serum albumin as a standard protein (Bradford 1976). We used the MDA–thiobarbituric acid (TBA) assay, which is used widely in studies of lipid peroxidation (Dahle et al. 1962). Serum (0.5 mL) from each subject was mixed with 1 mL of 10% (wt/vol) trichloroacetic acid, 0.8 mL distilled water, and 1 mL of 0.5% (wt/vol) TBA. After vigorous stirring, mixtures were incubated for 60 min in boiling water. After centrifugation at 6,000×*g* for 15 min, absorbance of the supernatant at 532 nm was measured and corrected for unspecific turbidity by subtracting the absorbance at 580 nm. The blank was corrected by addition of a 0.5% TBA solution in 10% trichloroacetic acid. We estimated the MDA concentration of each sample using a five-point standard curve with 1,1,3,3-tetraethoxypropane as an MDA standard.

### Separation of Se-containing proteins.

We separated Se-containing proteins using conventional chromatographic purification methods as described elsewhere (Deagen et al. 1993; Harrison et al. 1996; Mostert et al. 1998) with slight modification. Briefly, Se-containing proteins were purified using a tandem column system of two affinity chromatographic procedures. Serum (4 mL) was diluted with 4 mL equilibrium buffer of 0.02 mol/L ammonium acetate (pH 6.8), which was applied to a heparin-Sepharose column (1.0 × 10 cm^2^; Pharmacia, Uppsala, Sweden). The eluate was continuously passed through a blue-Sepharose column (1.0 × 10 cm^2^; Pharmacia). The eluate from the blue-Sepharose column contained the GSH-Px and was labeled fraction C. The fraction containing SeP (fraction A) was eluted from the heparin-Sepharose column with 500 U/mL heparin in equilibrium buffer. The fraction containing albumin (fraction B) was eluted from the blue-Sepharose column with 1.4 mol/L NaCl in equilibrium buffer at a flow rate of 0.5 mL/min. Protein profiles were monitored at 280 nm. Se and Hg concentrations in these three fractions and control buffer were determined by HG-AFS. Each fraction was then separated using sodium dodecyl sulfate-polyacrylamide gel electrophoresis (SDS-PAGE) as described elsewhere (Gao et al. 2003; Laemmli 1970).

### Statistical analysis.

Student’s *t*-test and multigroup comparisons of variables were carried out with one-way analysis of variance and the Statistical Package of Social Science (SPSS) for Windows (version 9.05; SSPS Inc., Chicago, IL, USA). Reported *p*-values were determined using a two-tailed *t*-test. *p*-Values < 0.05 were considered statistically significant. For bivariate analyses, we used linear regression.

## Results and Discussion

### Se and Hg in blood and urine.

Se and Hg concentrations in serum and urine samples analyzed by HG-AFS are shown in Table 1. The mean Hg concentration in serum of the exposed group was almost 40 times that of the control group, which was in agreement with the average background concentration of Hg. In urine of the exposed group, the mean Hg concentration was almost 75 times that of the control group. Among the exposed group, Hg concentrations in 12 subjects were extremely high, ranging from 67.5 to 210.3 ng/mL in serum and from 87.2 to 205.2 ng/mL in urine. The miners suffered typical symptoms: digestion dysfunction, hypomnesia, sleeping problems, tremor, and weight loss.

Although serum Se concentrations were significantly higher in the Hg-exposed group, the Se concentration in urine was only slightly higher and without statistical significance (Table 1). The molar ratio of Se to Hg was close to 1. The difference in the molar ratio between serum and urine concentrations of the Hg-exposed subjects was much greater than that in the control group. These results suggest that retention of Se increased in Hg-exposed individuals.

Total Hg concentrations in blood and hair are indicators of inhalation exposure to inorganic and methyl Hg vapor. In this study, we determined that exposure of local people and miners to Hg is due to not only inhalation of elemental Hg vapor but also to consumption of Hg-contaminated foodstuffs, which contained different Hg species. Even though the major source of Hg is elemental Hg, active transformation of inorganic Hg to organic Hg species (methyl Hg) was observed in water, sediment, and soil (Horvat et al. 2003). The average background concentration of Hg in urine has often been reported to be about 4 ng/mL in the general population, with an upper limit of about 20 μg/L (Agency for Toxic Substances and Disease Registry 1999; Iyengar and Woittiez 1988; Tsuji et al. 2003; World Health Organization 1990, 1991), whereas the normal range in serum is 1–8 ng/mL (Brune et al. 1991). The geometric mean concentrations of total blood Hg for women 16–49 years of age and children 1–5 years of age are 1.2 and 0.3 μg/L, respectively (U.S. Environmental Protection Agency 2001), which could be considered normal low levels for the general population. Thus, in the present study, the healthy control group from unknown contaminated areas also showed a normal Hg burden. However, the exposed group had high Hg concentrations, which was comparable with the data of the Mt. Diwata study in the Philippines (Drasch et al. 2001) and cases of Hg accidental poisoning (Hoeta and Lison 1997).

### Correlation analysis of Se and Hg.

**Figure 1:**
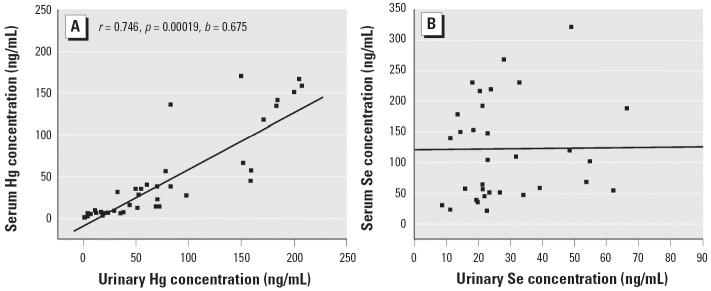
Linear regression analysis for Hg (*A*) and Se (*B*) concentrations in urine and serum samples from the Hg-exposed group.

**Figure 2:**
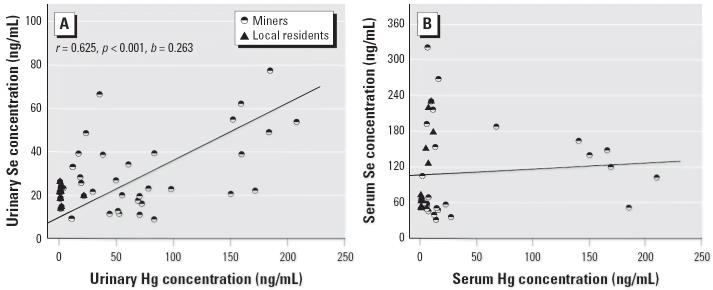
Correlation analysis of Hg and Se concentrations in urine (*A*) and in serum (*B*) samples from the Hg-exposed group.

There is a strong correlation (*r* = 0.746, *p* < 0.001) between Hg concentrations in serum and in urine of the Hg-exposed subjects, but there is no correlation for Se (Figure 1). A strong positive correlation is observed between Se and Hg concentrations in urine (*r* = 0.625, *p* < 0.001) but not in serum (Figure 2). It is reasonable that Hg excretion in urine is positively correlated with serum Hg concentrations. If serum Se and Hg concentrations are affected by short-term exposure, urinary Se and Hg concentrations may more accurately reflect their metabolism and excretion. Their correlation supports the interaction of Se with Hg *in vivo*. The results of our present study also suggest that Hg could interfere with the metabolic process of Se.

An earlier study by Hol et al. (2001) showed that the median concentration of Se in blood (119.2 ng/mL) was statistically significantly lower in subjects claiming to have symptoms of Hg amalgam illness than in healthy subjects with Hg amalgam but who had no symptoms of illness (130.3 ng/mL). Many studies indicated that retention and redistribution of Hg in experimental animal and fish were induced by administration of Se compounds (Bierregaard et al. 1999; Seppanen et al. 1998). It appears that the co-accumulated Se could alleviate the harmful effects of Hg exposure.

To our knowledge, the Se concentrations in individuals from the Wanshan area are not significantly higher than those in other areas (He 1996). Se concentrations in soil in the entire Guizhou Province range from 0.064 to 0.326 mg/kg, with an average of 0.369 mg/kg. In the Tongren District (including the Wanshan area), the Se content in soil of 0.15–0.20 mg/kg is considered relatively low (He 1996). The Se concentration in grain is slightly higher (unpublished data). No correlation between Hg and Se in rice has been found; therefore, Se probably does not play a role in Hg uptake and retention in rice grain (Horvat et al. 2003). Moreover, it is interesting to note that the average Hg concentrations in Guizhou fish liver and muscle samples are, respectively, 25 and 5 times those of Hg concentrations in samples from individuals exposed to low concentrations of Hg. Se content in both liver and muscle samples from fish showed no significant differences (Zhang et al. 2004). Zhao et al. (2004) found that Hg exposure in pigs causes redistribution and reaccumulation of Se and Hg in tissues and various corresponding organelles. The main sources of food for both the exposed and control groups are rice, fish, and vegetables. Therefore, Se intake in the exposed subjects may be only slightly different from those of normal controls. Nevertheless, our results suggest that the increased retention of Se was associated with elevated Hg burdens in the Hg-exposed subjects. It could be deduced and confirmed that Hg exposure affected bioavailability and retention of Se in humans.

### Hg and selenoproteins in serum.

**Figure 3:**
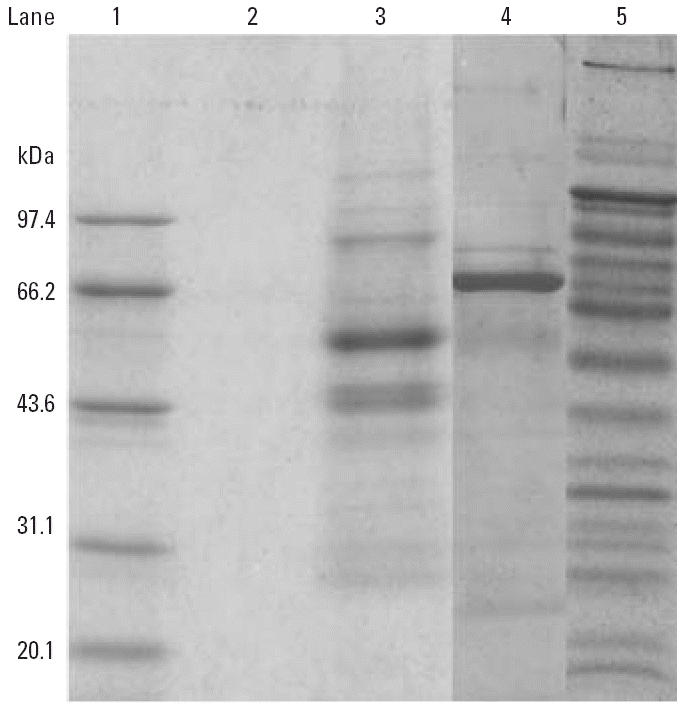
Coomassie-stained SDS-PAGE gel for serum proteins after separation by heparin-Sepharose and blue-Sepharose affinity chromatography: lane 1, protein standards (from top to bottom: 97.4, 66.2, 43.6, 31.1, 20.1, and 14.4 kDa); lane 2, sample buffer; lane 3, fraction A (mainly SeP); lane 4, fraction B (mainly albumin); lane 5, fraction C (containing GSH-Px).

Hg and Se concentrations in various protein fractions separated from serum by heparin- and blue-Sepharose affinity chromatography were analyzed by HG-AFS. Se concentrations associated with GSH-Px and SeP were 2 times higher in the Hg-exposed group than in the control group (Tables 2, 3). Thus, expression of both SeP and GSH-Px was highly up-regulated in Hg miners and was accompanyed by increased serum Se concentration. As shown in Figure 3, proteins of fractions A (SeP) and C (albumin) are almost purified because of the highly efficient effect of affinity chromatography. The band of fraction C showed a molecular weight of 66 kDa for albumin. The two major bands in fraction A are two isoforms of SeP with molecular weights of approximately 58 and 49 kDa. In fraction B, the staining gel indicated a number of protein bands. Therefore, affinity chromatography can give only an estimate of plasma Se in selenoproteins.

Although Se concentrations in serum of exposed and control groups are different, the fraction of Se entering SeP is approximately the same, ranging from 45 to 62% of total Se exposure, which is in agreement with other published data (Harrison et al. 1996; Plecko et al. 1999). The percentage of nonspecific binding of Se with albumin decreased, and the percentage of Se bound to GSH-Px increased from 28.7 to 33%. The Sec residue in selenoproteins as GSH-Px and SeP is co-translationally incorporated via a predefined UGA codon, which has a specific covalent binding capacity. The Se concentration in the albumin fraction was approximately the same regardless of total Se exposure, which suggests that the nonspecific Se-binding capacity of albumin is stable and the remaining Se goes into the selenoprotein pool.

We found that Hg concentrations in various protein fractions of the control group were very low and close to the detection limit. We also found that a small fraction of Hg was bound to serum albumin (Table 3). Most Hg was present in the fractions containing SeP and GSH-Px. The SeP-containing fraction bound more Hg, whereas the Se:Hg molar ratio was much lower in the exposed group than in the control group (Table 2). These results indicate the strong interaction of SeP and Hg at high Hg exposure concentrations; at Hg exposures too low to affect the Se status, this interaction has not been reported (Falnoga et al. 2002).

Other studies have found that pure SeP binds with Hg, but our results do not support this finding. Although SeP is the main protein found in fraction A, a minor protein impurity may be associated with Hg. Generally, the highly purified SeP was obtained using immobilized monoclonal antibodies (Himeno et al. 1996) or multistep separation involved in heparin-affinity, anion-exchange, and metal-chelate affinity chromatography (Mostert et al. 1998). The process, with high salt concentrations and metal-chelate agarose, may result in Hg desorption from its binding proteins during the separation process. Monoclonal assays for SeP and GSH-Px in plasma are not commercially available, which makes them expensive to produce in the laboratory. This has hindered progress on the interaction of SeP and Hg.

SeP is presumed to be involved in alleviating the toxicity of heavy metals (Whanger 2001) and it is known that SeP is preferentially absorbed by the brain but not by other organs in Se-deficient animals. Although Se counteracts the neurotoxicity of Hg, cadmium, lead, and vanadium, the detoxification mechanism is unknown.

Results from animal studies have shown that Se reacts with Hg in the bloodstream by forming complexes (Yoneda and Suzuki 1997). SeP binds Hg and Se only when Hg^2+^ and selenide are present simultaneously and does not bind Hg^2+^ or selenide separately in plasma (Suzuki et al. 1998). The complex binds selectively to a plasma protein, SeP, to form an [(Hg-Se)*_n_*]*_m_*–SeP complex up to *n* = 100, *m* = 35 *in vitro* (Sasakura and Suzuki 1998; Suzuki et al. 1998). The results of our present study indicate that the percentage of Hg bound to SeP increases with increasing Hg concentrations in serum, which represented a metabolic process in humans.

The cDNA sequence of SeP contains 10 Sec, 17 Cys, and 28 histidinyl residues, indicating that SeP is capable of binding a metal via these amino acids. The toxicity of Hg is associated with its reaction with sulfhydryl groups of Cys in proteins (e.g., metallothionein and a variety of enzymes) and thiol-containing small molecules such as Cys and reduced GSH *in vivo*. The selenol group shows even stronger affinity for Hg than for the thiol group because the much lower p*K*_a_ of Sec (~5.4) gives it a higher reactivity than Cys (~8.0). Therefore, the properties of SeP containing 6–10 Sec groups and the corresponding high reactivity of its selenol groups increase both the possibilty and capability of binding more Hg. Although albumin has more than 30 cysteinyl residues, its Hg-binding ability is limited, which is confirmed by our results. Thus, at very low Hg concentrations in serum, Hg may bind to a variety of different protein fractions, whereas the stronger competition and reactivity of the selenol group in selenoproteins explains why more Hg was observed in the SeP-containing fraction at higher Hg exposure concentrations.

### Oxidative status in serum.

**Figure 4:**
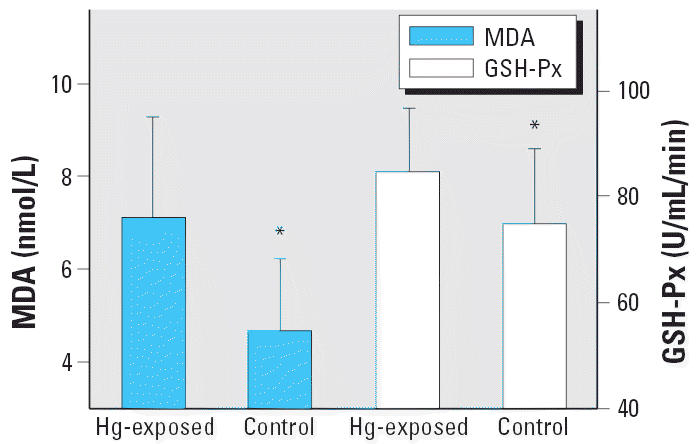
The MDA concentration and GSH-Px activity in serum samples of the Hg-exposed people and control. Data are expressed as mean ± SD. *p < 0.05.

Activity of GSH-Px in serum of miners was significantly higher (*p* < 0.05) than that of the control group (84.6 ± 12.1 vs. 75.0 ± 14.1 U/mL/min). The lipid peroxidative product MDA increased significantly in the Hg-exposed group (7.12 ± 2.18 vs. 4.69 ± 1.55 nmol/mL, *p* < 0.05), as shown in Figure 4. The positive correlation between total Hg concentrations and MDA concentrations in serum was also observed in the present study (*r* = 0.568, *p* < 0.05). The oxygen-derived free radicals can induce the lipid peroxidation reaction of multiple-valent unsaturated fatty acids in plasma, which ultimately produces lipid peroxidation products such as MDA. Therefore, the MDA concentrations reflect the degree of oxidative injury *in vivo*. Although the results of the present study suggest that oxidative damage existed in the Hg-exposed individuals, exposures to other environmental contaminants may also contribute to elevated MDA concentrations.

The antioxidative properties of selenoproteins have attracted increasing interest. Extracellular GSH-Px reduces hydrogen peroxide and *t*-butyl hydroperoxide and shows some activity against phospholipid hydroperoxide. Although SeP reduces phospholipid hydroperoxide, it has no effect on hydrogen peroxide and *t*-butyl hydroperoxide. SeP is also reported to function as a peroxynitrite scavenger or cell survival factor in primary cultures of neurons (Yan and Barrett 1998). The imbalance of redox status, especially profound oxidative DNA damage, was found among Hg-exposed individuals (Chen et al. 2005). In the present study the significantly elevated MDA in serum indicated oxidative stress induced by Hg exposure. However, the elevated antioxidant enzymes or proteins such as GSH-Px and SeP could eliminate the increased reactive oxidative species. Thus, we propose that selenoproteins may have two important roles in protecting against Hg toxicity. First, they may bind more Hg through their highly reactive selenol group, and second, their antioxidative properties help compromise the reactive oxygen species induced by Hg *in vivo*.

## Figures and Tables

**Table 1:** Se and Hg concentrations (ng/mL) in serum and urine samples from Hg-exposed and control groups.

	Hg-exposed group (*n* = 37)		Control group (*n* = 35)	
Samples	Se	Hg	Se	Hg
Serum				
Mean ± SE	100.9 ± 87.6	38.5 ± 61.5	67.2 ± 18.2	0.91 ± 0.28
*t*-Test	*p* < 0.01	*p* < 0.01		
Range	13.3–220.5	1.85–210.3	40.4–84.8	0.41–1.20
Molar ratio (Se:Hg)	6.7 ± 3.6		175.5 ± 74.6	
Urine				
Mean ± SE	24.7 ± 16.5	86.8 ± 65.2	22.2 ± 10.1	1.25 ± 1.5
*t*-Test	*p* > 0.05	*p* < 0.01		
Range	8.78–76.3	11.0–205.2	10.2–34.5	0.3–2.4
Molar ratio (Se:Hg)	0.7 ± 0.6		45.3 ± 17.2	

**Table 2:** Se and Hg concentrations (ng/mL serum ± SD, *n* = 12) in SeP (fraction A) after affinity chromatographic separation of serum from Hg-exposed and control groups.

Subjects	Se in SeP	Percent Se in total serum	Hg in SeP	Percent Hg in total serum	Molar ratio (Se:Hg) in SeP
Hg exposed	59.7 ± 11.2	55.2 ± 6.4	19.4 ± 9.2	33.4 ± 15.9	7.8 ± 3.1
Control	31.5 ± 10.2	52.5 ± 7.9	0.15 ± 0.12	15.4 ± 8.2	535 ± 216
*t*-Test	*p* < 0.05	*p* > 0.05	*p* < 0.05	*p* < 0.05	*p* < 0.05

**Table 3:** Se and Hg concentrations (ng/mL serum ± SD, *n* = 12) in Se-containing proteins after affinity chromatographic separation of serum from Hg-exposed and control groups.

	Hg-exposed group		Control	
Fractions	Se	Hg	Se	Hg
Fraction B (mainly albumin)	12.7 ± 5.8	2.2 ± 1.2	11.3 ± 4.9 (*p* > 0.05)	0.24 ± 0.18
% in total serum content	11.7	6.6	18.8	24.7
Molar ratio (Se:Hg)	14.7		119	
Fraction C (containing GSH-Px)	35.7 ± 12.7	45.8 ± 15.4	17.2 ± 7.8 (*p* < 0.05)	0.58 ± 0.25
% in total serum content	33.1	60.5	28.7	59.8
Molar ratio (Se:Hg)	1.98		75.5	
